# Control Matters in Elder Care Technology: Evidence and Direction for Designing It In

**DOI:** 10.1145/3532106.3533471

**Published:** 2022-06-13

**Authors:** Clara Berridge, Yuanjin Zhou, Amanda Lazar, Anupreet Porwal, Nora Mattek, Sarah Gothard, Jeffrey Kaye

**Affiliations:** University of Washington; University of Texas at Austin; University of Maryland; University of Washington; Oregon Health & Science University; Oregon Health & Science University; Oregon Health & Science University

**Keywords:** Health technology, Remote monitoring, Assistive technology, Privacy, Control, Aging, Older adult, Dementia, Memory loss

## Abstract

Studies find that older adults want control over how technologies are used in their care, but how it can be operationalized through design remains to be clarified. We present findings from a large survey (n=825) of a well-characterized U.S. online cohort that provides actionable evidence of the importance of designing for control over monitoring technologies. This uniquely large, age-diverse sample allows us to compare needs across age and other characteristics with insights about future users and current older adults (n=496 >64), including those concerned about their own memory loss (n=201). All five control options, which are not currently enabled, were very or extremely important to most people across age. Findings indicate that comfort with a range of care technologies is contingent on having privacy- and other control-enabling options. We discuss opportunities for design to meet these user needs that demand course correction through attentive, creative work.

## 1 INTRODUCTION

Older adults and people living with dementia are among the most targeted groups for technological intervention in the name of risk assessment and management [7, 59, 136]. The desire to predict and prevent health events through increased remote monitoring and analysis of behavioral biometrics is driven by health and long-term care cost containment and the widespread preference to manage chronic conditions at home. The need to be attentive to older adults’ preferences for control and privacy enabled in technology design is pressing because of the strong momentum in many countries to rely on technologies in care provision, without fully considering how they could restrict rather than enhance autonomy, privacy, human connection, and other important needs and values [14, 17, 20, 51, 60, 62 In addition to potentially restricting important needs and values, the ways that these emerging technologies instantiate control and privacy may impede adoption. Research has found that negative attitudes that older adults hold towards technology stem in part from concerns about technology interrupting home life, as well as privacy threats [15, 101].

Technology acceptance research has dominated the field of gerontechnology with little attention to the assumptions participants make about control options they might have or that they might require. The field of human-computer interaction (HCI) and design practice are ideally positioned to address this gap. Human-computer interaction researchers are establishing the importance of control and privacy for older adults, primarily through qualitative research [51, 62, 95, 144]. This project takes the approach of survey research, gathering feedback from older adults about the elements of control that are important to them in care technology. With the growing recognition that the category ‘older adult’ represents an extraordinarily diverse group of people, surveys are useful for breaking down how control needs may differ between people along certain characteristics. For example, people with memory concerns, mild cognitive impairment (MCI), and dementias are often targeted for remote monitoring – yet it is unknown how their preferences compare to others’ [24].

We conducted a large (n=825) survey with an online U.S. cohort to better understand potential users’ perspectives on various options that enable control over elder care technologies. With an eye to designing for the future, the sample is age diverse, while skewing older than age 64 (n=496), so that we can compare across age as well as deepen our understanding of the preferences of current older adults. We engaged in this work to identify opportunities to “design in” control and to identify potential disconnects where more attentive design visioning is needed. Following research on the control desired by older adults in the context of monitoring technologies, we conceptualize control as a form of ongoing agency in which an individual can decide if and what information is collected about them and with whom it is shared [111, 149]. Survey participants were first presented with three distinct types of data collection and use: location outside of the home, audio for detection of changes to brain health, and visual with audio telepresence robot (video chat on wheels). After asking them to assess their comfort level with each, we presented five control-enabling options, which gerontechnology domain experts have identified as ways to mitigate prominent risks posed by these and other technologies used in elder care [18]. Survey participants rated the importance of each option that could be applied to these particular technologies and beyond.

Our findings show that across the many health and sociodemographic factors that we accounted for, people place high importance on being able to exercise basic control over technologies that could be used in their care. Comfort was relatively high with a wide scope of types of data collected and transmitted for use by one’s primary support person,^1^ but comfort is contingent on basic control options that are not standard options in the design of elder care technologies. This paper contributes actionable evidence, including the value of operationalizing these forms of control, and provides specific design recommendations to better match the needs of older adults. In doing so, our work advances the growing body of research that aims to counter ageist stereotypes in design [146], rebalance unfavorable power dynamics in care, and mitigate other risks of data-intensive technology use.

## 2 BACKGROUND AND RELATED WORK

Technologies to support older adults and their caregivers at home is an area of strong growth for design. Policy makers, providers, and family caregivers are looking to technological solutions and investing in artificial intelligence (AI) and other technologies that monitor activity and safety of older adults, with a particular focus on Alzheimer’s disease and related dementias (e.g., the AAL Joint Platform of the European Union and AGE-WELL Initiative in Canada) [25, 69, 91, 127, 129, 138]. In the U.S., health care payment systems including Medicaid are now beginning to cover a range of technologies that have remote monitoring functions [17, 28], foretelling significant growth as lack of third party reimbursement had impeded startups in the aging space [19, 129].

The nature of data continuously collected about older adults is also rapidly changing, especially as big tech has recently established itself in the space of monitoring and risk assessment of older adults [81, 151]. There is particular excitement about the potential capability of detecting cognitive change using predictive linguistic markers [18, 112], which involves audio capture in the home. Location tracking is becoming ubiquitous [25, 48], and the problem of social isolation during COVID is further spurring the evolution of telepresence [64, 87]. These three categories of technologies represent distinct types of data collection that may be carried out passively with little room for direct control by the older adult.

Sustained focus on the control needs and preferences of older adults in relation to these and other monitoring technologies has been delayed, in part due to what has been termed by Peine and Neven the ‘interventionist logic’ that has dominated and which problematizes old age and prioritizes the needs of caregivers [114, 115, 146]. This dominant logic together with the marginalization of issues of elder care in a range of fields that are concerned with power, privacy and other values in sociotechnical practices, such as HCI, science and technology studies (STS), and surveillance studies [51], has left us with limited and vague understanding of the specific controls desired by older adults in technologies used for care purposes. This gap in knowledge and inattention to older adults’ needs for control over how they are monitored may set up technology-mediated care practices to fail or disempower.

### 2.1 What’s at stake for older adults in how technologies are used in their care?

Invasion of privacy is by far the most often cited threat posed by elder care technologies that older adults are concerned about [14, 20, 47, 51, 89, 113, 132, 155, 157]. Older adults in general tend to have greater privacy concerns about Internet use and fraud but lower privacy self-efficacy than do younger adults [8, 156]. Research also indicates that privacy needs and concerns will vary with the technology and data collected [20, 128], meaning that personalized control options would be worthwhile design elements. Privacy law scholar, Cohen’s conceptualization of privacy interests as “an interest in breathing room to engage in socially situated processes of boundary management” [35:^149^] is useful to this context of elder care because it foregrounds the fact that privacy is necessary for individuation as it relates to subjectivity, and that one’s ability to manage these boundaries is protective of “the capacity for self-determination” [36:1905]. To have a degree of control over how one is monitored is to be able to engage in boundary management and be protective of personal privacy needs [14].

Privacy is inextricably linked to other values. It enables values that older adults tend to care about, like freedom and autonomy [14, 20]. HCI researchers, McNeill and colleagues draw similar conclusions about the important role of privacy [96]. They provide a privacy framework that includes functions of privacy identified through interviews with older adults. These functions are Self-protection, Autonomy, Emotional release, Confiding (“control over the extent of information disclosed and to whom it is disclosed”), Social identity, Self-concept, and Protecting others. The authors recommend autonomy-enabling options, such as control over when a device is collecting data, and caution that “if AAL technologies are a success at the expense of the individual’s privacy then to what extent is the AAL really empowering or improving the well-being of the elderly?” [96:^101^].

Risks have been associated with monitoring technologies used in dementia care in the home, including location tracking outside of the home, audio recording, and roaming telepresence. These risks include the fact that privacy invasion for the individual and for visitors can cause self-limiting activity or diminished autonomy, independence, and agency, and can compromise dignity through information capture (including inferred) about sexual or bodily functions [18]. Risks also include caregiver overreach and unnecessary harassment based on data, as well as feeling infantilized or “baby sat,” uncomfortable at home, without a place to hide, bugged, and uneasy if a caregiver could visually or audibly enter at any time. This could lead to distrust, suspicion, anxiety or paranoia [18]. Of primary concern is also the risk that the use of these data could enable fewer social calls or substitute for personal visits. Caregiver stress is also cited as a risk should they receive ambiguous data, not understand what warrants an intervention, or experience information or alert overload [18].

### 2.2 What do we know about older adults’ preferences for control?

There is little in the gerontechnology literature on specific desirable options to enable control. Much of it focuses on data flows, such as willingness for in-home monitoring technologies to share personal and health information [13, 24, 68, 73]. Acceptance tends to be high with regard to sharing health information with medical providers or family members, but older adults may express distrust in digital technologies with diffuse data implications and take a moral position against the loss of privacy as a cultural value [74]. Values dissonance can contribute to resistance to use of new technologies [12, 15] and uncertainty or not understanding a technology or its data flows begets distrust [74].

Qualitative studies have found that control tends to be very important to older adults [20, 44, 52, 111]. Small studies have shown that they want control over decision making about what data are accessed by whom under what conditions [46, 52, 89, 149]. Mean-while, systems that enable control by older adults are not the norm, and many that have a monitoring function are fixed or hard to customize to accommodate personal preferences [60, 105, 145].

The work in HCI on the needs of older adults has been expanding [12]. In a study of 12 people in care facilities about the sharing of health and well being data with caregivers, Nurgalieva et al. found that control mattered to residents who wanted to maintain it as long as possible - even in these spaces where privacy is more limited than it is in home and community environments [111]. The authors conclude that “designers should ensure that information exchange occurs with informed consent and is aligned with seniors’ preferences for privacy and control” [111]. A qualitative study of home robots with 30 older adults found that the need for control was one of four user needs that robots potentially threaten [44]. Another small-scale study of older adults’ preferences for companion robots found that control was a significant factor for some, which factored into their preference for an animal form because having a pet-like robot conveys a sense of control over it [34]. Control was found to be “a key condition for privacy preservation” by Schomakers et al. when they surveyed 97 adults, including many over the age of 50, about fall detection and vital parameter monitoring [128]. The authors explain that privacy concerns “do not only concern information privacy and data security, but all dimensions of privacy are touched as intimate and private aspects of life are digitized and the physical, psychological, and social self is made more available to others than desired” [128]. Their survey respondents reported that they desired control over what, how, when, where, and to whom data flows [128]. Frik and colleagues [51] similarly conclude, based on an interview study with 46 older adults, that designers must improve transparency and control as part and parcel of addressing misconceptions among older adults about data flows.

Extant research suggests that empowering older adults with control over data-intensive health and elder care technologies will only become more important as use climbs and populations age [73]. This will require a finer-grained understanding of how to enable control and its prerequisites, such as awareness, and to learn what options might be important to which subgroups of older adults for whom products are designed to support.

### 2.3 On power and why who’s given control matters

Due to its surveilling nature, elder care monitoring technologies used in the home are ripe for familial conflict and stress [20, 70]. Studies have found differences between older adults and family members with regard to perception of need and comfort with the collection of various levels of data granularity [16, 20, 58, 89]. Older adults and adult children are likely to have different preferences because the risks and benefits of monitoring fall differently to each [20, 54, 110]. A qualitative dyadic study of Meals on Wheels clients and their adult children found that adult children rated passive remote monitoring technologies more favorably than did their older parents and expressed conflicting views with those of their parents about how and when they should be used [20]. Adult children overwhelmingly underestimated their parents’ demonstrated ability to comprehend the basic functions of these technologies and thought they would engage them minimally in decisions about adoption [20].

Given the potential risks and ‘intimate threats’ [84], it is especially important to take a close look at power dynamics that are enabled, amplified, or disrupted through those technologies. Critical data studies scholars have emphasized that ethics discourse and research must incorporate explicit attention to power [27, 61]. This is of particular relevance to the design of technologies for elder care [94].

Burmeister and Kreps write that “Rather than allowing power to be exercised in an intuitive or unconscious manner” [27], design needs to not just be attentive to power, but to design intentionally with it top of mind. This is particularly important because older adults are likely to assess their own risk differently from how others assess it such that proxy decision making may not reflect the decisions older adults would otherwise make for themselves [125]. The practices that develop around remote monitoring can negatively impact older adults where greater exercise of power over aspects of their lives is authorized [16, 54]. For instance, an ethnography of dementia care dyads revealed troubling ways in which using sensors and fall detectors led caregivers to restrict movement and remove privileges, as well as reduce their personal visits [79]. Ethicists and gerontologists have cautioned that older adults may be vulnerable to infringements on self-determination through remote monitoring [15, 60, 106], as well as the ways in which decision making based on AI monitoring “may inadvertently intensify power and control” [60:^3^].

### 2.4 The need for analysis of potential difference

Previous research has found that age, formal education, gender and sexual identity, race and ethnicity, and health conditions are factors that could affect comfort and willingness to share personal health or other data use in remote monitoring [2, 13, 26, 50, 56, 65, 72, 75, 118, 141, 153]. A common assumption made about future generations of older adults when considering study findings about current older adults’ privacy concerns is that those concerns will essentially age out because younger older adults will hold very different expectations for privacy. That is, they’ll be inured to more extensive data collection about them. Because different future user needs would have significant implications for design practices, it is vital that these assumptions be tested. Further, despite the heterogeneity of people over age 64, research on technology preferences in HCI often lacks insight into potential differences among older adults by socio-demographic and health characteristics [118]. Research shows us that older adults are heterogeneous in terms of tech use [39] and that views vary greatly by individual for perceived benefits and privacy risk [128], but the heterogeneity and fine-grained nature of older adults’ privacy preferences are understudied [50, 146]. Designing for a non-homogenous group thus requires a closer look at socio-demographic characteristics, acknowledging that different preferences and needs may be present based on lived experience in different body-minds with accumulated diverse life experiences. For example, Poulsen et al. found that LGBTIQ+ older adults had specific concerns about their vulnerability to robot data security, indicating a greater perceived vulnerability to personal data collection [118]. Learning more about such potential differences would therefore provide valuable insights into how socio-demographic factors may interact with preferences for control over elder care technologies.

It may be particularly important to understand the needs of people who are concerned about their memory or other possible signs of dementia–not only because so much technology is designed for dementia care use–but also because gerontology research has demonstrated various ways in which the preferences of people living with dementia can go unrepresented [23, 57, 98, 100, 152]. Those who provide care for this large, marginalized group of people are increasingly looking to technologies to support caregiving. Studies show that people living with dementia often prefer to be more involved in decision-making than they are [100], and that losses in autonomy with a dementia diagnosis are stressful to them [134]. These stressors may be compounded if they do not have a sense of control over how data-intensive technologies are used in their care.

### 2.5 Risk mitigation by design

What can design do to enable feelings of control, privacy preservation, and to otherwise mitigate the risks that accompany elder care technologies that can be experienced as intrusive? And how can design create the most relevant control-enabling features possible for a person living with dementia? Enabling control for diverse needs of older adults will take extra design work. First, beyond knowledge that older adults would generally like to maintain as much control as possible, a clear understanding of what specific forms of control are desired is needed. Understanding more concretely what options would be valued is key to mitigating risk through design.

Researchers within and adjacent to HCI have offered responsive, creative suggestions for accommodating the needs of older adult users [51, 55, 96, 145, 149], including some focused on those living with dementia [95, 123]. Often, authors infer design implications and suggestions from needs identified in small-scale studies. Close, attentive qualitative design research such as these, and particularly those engaging people living with dementia, produce rich insights and ways of understanding [80, 104]. Our relatively large-scale survey study takes a next step in the process of understanding how to design more empowering elder care technology.

## 3 METHODS

This study involved a survey completed by 825 individuals. Rather than ending with design implications inferred from project engagement with older adults, we have fed specific user options back to a large number of older adults so we can learn directly from them if they would indeed be worthwhile. Further, due to the large sample size, we are able to offer unique insights into ways in which people might differently weigh the importance of control options by their age, gender, formal education, having a parent with dementia, and self-reported memory status, among other characteristics.

The survey was based on a review of the literature and our findings from a prior survey process, which was a Delphi study of interdisciplinary domain experts [18]. The control options that were derived from domain experts in this Delphi study are tightly linked to risk mitigation but, like most studies, they are not generated directly by older adults, nor have they been given the opportunity to vet them. The survey study reported in this paper completes this feedback loop to assess the importance of these control options in order to better guide design and future user studies.

### 3.1 Survey design

We created a survey consisting of 19 items organized by three sections: technology scenarios, options, and artificial companionship. In this paper, we focus on the responses to the scenarios and options sections (see the survey in Appendix A.1). Content for the current survey was drawn from a review of the literature on risks and benefits associated with remote monitoring technologies, as well as from the findings of our published multi-wave Delphi expert survey on dementia care technologies used in the home [18]. That multi-wave survey was conducted with domain experts in aging and technology research, design, and implementation in the U.S. and Canada. It included disciplinarily diverse participants, such as social scientists in gerontechnology. The Delphi study was conducted to identify technologies that will be prevalent in home dementia care in five years, along with their benefits, risks, and risk mitigation strategies, and was informed by a review of the state of knowledge on near-future home-based technologies for dementia care [18]. The study used the Delphi approach to obtain excerpts’ opinions and build consensus with them regarding their responses. We selected this expert study as the foundation of our survey to respond to gerontechnology researchers’ call for design and development to be more informed by gerontological and social sciences knowledge [39, 40, 42, 49, 66, 88, 129]. In this way, this study puts gerontology into more direct conversation with HCI.

The survey presented in this paper draws on the technology scenarios and options developed in our prior work [18]. For the technology scenarios, the expert study had participants identify the technologies they predicted would be the most prevalent in dementia care in the home in the next 5 years (see [18]). We selected three of the twelve technologies identified by experts for the current study: location tracking outside of the home for safety, in-home speech/audio analysis for early detection of brain changes, and video and audio capture through telepresence on wheels. We chose these three technologies because they are each 1) among the most commonly endorsed by domain experts as likely to cause tension and require a conversation with the older adult and caregiver before use, and 2) each represent a distinct type of data collected by a range of elder care technologies that, together, represent a range of data types: location, visual, and audio; and 3) each can be communicated with confident clarity in a self-administered online survey format. For the purpose of this survey, we created scenarios around the three technologies and added a time variant for the telepresence robot (video chat on wheels) for pandemic vs. normal times (see Table 2).

Our survey also presents five control options derived from the Delphi study, where experts produced ideas for how to mitigate risks that they had identified. The five control options we chose for the current study are the most commonly endorsed risk mitigation strategies from this past work [18]. These risk mitigation strategies are:

Devices should actively remind users of how they operate, who is controlling them, and how data are being collected and used
Establish informed consent as a process
Allow person living with dementia to stop or not use
Allow repetitive opportunities to try it out, decline to use, and try again
Avoid continuous streaming—require permissions for video or audio
Enable ability for person living with dementia to pause system/data collection

We translated these expert-identified risk mitigation strategies into five specific, actionable user options for our survey (see Table 4) to make them concrete and understandable for survey participants (i.e., non-domain experts). These five options align well with extant work in HCI. For example, both [145] and [51] have illustrated the need for feedback about what data are being collected about older adults in order to clear up potential confusion about data flows and enable informed use. While the five options do not represent an exhaustive list of control options that may matter to older adult users, they do act as both a drilling down and synthesis of many of the suggestions put forward by both ethics and HCI researchers [95, 96, 123, 145].

### 3.2 Study participants

The survey was administered using Qualtrics and disseminated by email in June of 2020 to a pre-existing online survey cohort of the Research via Internet Technology and Experience (RITE) program of the Oregon Health & Science University (OHSU).^2^ The only inclusion criteria for this cohort is to be over the age of 18. The volunteer RITE Online Cohort was recruited using OHSU’s Oregon Clinical and Translational Research Institute’s Cohort Discovery and through social media campaigns and flyers. Social media campaigns and flyers were secondary recruitment strategies. Health and socio-demographic data are collected as part of intake and are updated through an annual survey.

The current study’s survey was distributed online to all 2,434 members of the RITE cohort. We achieved a response rate of 45% (1,082). Because the focus is on technologies used in the home setting, two respondents living in assisted living were excluded for analysis. Those without data for key variables of interest, age (missing=4), gender (missing=72), education (missing=150), or memory problem history (missing=179) were excluded, leaving a final sample of 825. The acceptable rate of missing value for each covariate of secondary interest ranges from 0.1% to 4.2% and 9.1% for the variable history of dementia in parents.

Table 1 presents the health and socio-demographic characteristics of our sample. Compared to the general national population, the study sample is older, whiter, and more formally educated. The respondents’ ages range from 21–92 years with a mean of 64 (SD=13.13). Sixty percent of respondents are 65 or older while only 16.5% of the general U.S. population is 65 and older [142]. Sixty-six percent of this sample are women. The majority of respondents (74.2%) have a college degree or more education–far higher than the 32.1% of the U.S. general population. Ninety-six percent of respondents are white, compared with 76.3% of the population [142]. Because our sample skews older than the general population, nearly one quarter (24.4%) of our sample report either current memory problems and/or that they have been seen by a physician for memory problems, which is a far greater percentage than the general population. The percentage of those within our sample over 65 who report memory problems is consistent with most population studies [37, 38, 147].

This sample is also far more technologically experienced and resourced and may thus represent a group that would be expected to be early adopters of new technologies. The vast majority of our sample (84.3%) rated their confidence using the computer as very high. Ninety-five percent of our respondents report using the computer daily while 81% of the general population reports going online daily [117]. Our sample also differs from the general population in their greater access to wireless internet (95% vs. 77%) [116]. Only 75% of the general 65+ population uses the internet [116] and 42% do not have wireless broadband at home [63]. While our sample skews older, among our participants 65+, 93.3% have wireless internet and 100% use the internet. These sample characteristics are discussed further in the limitations section.

### 3.3 Dependent variables

Comfort was assessed for three distinct types of data collection and use: location tracking while driving for safety, in-home speech/audio analysis for early detection of brain changes, and video and audio capture through telepresence robots. The four response options are Very Uncomfortable; Somewhat Uncomfortable; Somewhat Comfortable; Very Comfortable. For the telepresence technology, described as video chat on wheels, participants were asked two questions about their comfort level: in scenarios of “during normal circumstances” and “in unusual times when someone cannot come to your home such as during the coronavirus pandemic.”

The importance participants place on technology control options was assessed through five questions (presented in Table 4). The six response options are Not at all Important; Very Unimportant; Somewhat Unimportant; Somewhat Important; Important; Extremely Important. As described in section 3.1, these options were derived from the risk mitigation strategies recommended by gerontechnology domain experts for data-intensive technologies used in dementia care in the home (see [18]).

### 3.4 Independent variables

Sociodemographic characteristics and personal health conditions were pre-collected through the RITE cohort surveys. According to prior literature, characteristics that have been shown to be associated with comfort with technologies were used in bivariate and multivariate analysis, including age [140], gender [29], education [78], marital status [1], living alone or with others [78], confidence using computers [68], social support [9], number of chronic conditions [78], and memory problem history [30, 39]. The Brief Assessment of Social Engagement (BASE) scale ranging from 0–20 [103] was used to assess engagement in social activities outside of the home as one indicator of social engagement and support, including travel out of town, attending religious events, attending clubs or group events, visiting friends/family, and eating out. Each item of the BASE scale was rated on the basis of frequency (0 rarely or never, 1 yearly, 2 monthly, 3 weekly, 4 daily). Respondents were asked if they had self-reported current memory problems and if the participants were seen by a physician for memory problems. A dichotomous variable, memory problem history, was positive when a respondent replied yes to either of these two questions. Having a parent with a history of dementia was also included because experience with dementia could impact assessment of the technology scenarios or options and it might be relevant to respondents’ perceived risk of acquiring dementia [71]. The limited response options for this cohort’s pre-collected gender question are a limitation of this study that resulted in the exclusion of six respondents, which we discuss in the limitations section.

### 3.5 Analysis

Descriptive analysis was conducted using R software [120]. We conducted bivariate and multivariate ordered logistic regression [22] using R package “MASS” [121] and “ordinal” [31] to examine the associations between independent variables and respondents’ comfort with three types of technologies. The Wilcoxon Signed-rank test [154] was used to detect differences in responses to the question about comfort level with video-chat on wheels in normal vs. pandemic times.

We used latent class analysis (LCA) to identify classes of respondents reporting similar patterns of their perceived importance of technology control options. This “person-centered” approach allows us to identify complex patterns or typologies of participants in multivariate categorical data [130]. The five ordinal technology control options questions were used to classify respondents into subgroups based on the differences and similarities of their responses to these five questions. Some existing R packages including poLCA [86] performed LCA for polytomous nominal categorical variables. As treating ordinal variables as nominal excludes potentially useful information contained in those variables, we modeled the ordinal nature of responses using an adjacent category logit model [4, 5]. We estimated parameters of the model using Expectation Maximization (EM) approach [43]. The optimal number of classes was determined using the Bayesian information criterion (BIC) to balance model fit and parsimony [130]. We then generated a jittered spaghetti plot of respondents stratified by their estimated latent class. Detailed descriptions of the LCA analysis process are provided in Appendix A.2.

After we determined the number of classes, we examined the associations between independent variables and classes using the Welch one way test [102] for independent variables that are continuous variables (age and social activity level scores) and Fisher’s exact test [3] for independent variables that are categorical.

## 4 FINDINGS

### 4.1 Comfort with technology scenarios by health and demographic characteristics

Response frequencies to each of the four technology scenario questions are presented in Table 2. 71.1% (561) felt somewhat or very comfortable if their primary support person tracked where they are when they are driving to make sure they are safe. 57.9% (478) felt somewhat or very comfortable with their primary support person recording audio in their home to learn if and when they might be experiencing changes in their brain health. 73.3% (605) felt somewhat or very comfortable with video chat on wheels driven by their primary support person in their home during pandemic times and 62.1% (512) felt this way under normal circumstances. Respondents were significantly more comfortable during pandemic versus normal times (p<0.001) with video-chat on wheels; however, the effect size (0.19) indicates that this statistically significant difference is small.

Table 3 presents those characteristics with statistically significant relationships to reported comfort with each of the three technologies, including bivariate results. In bivariate analysis, higher age by one year (odds ratio [OR] = 1.01; 95% confidence interval [CI] = [1.00, 1.02], p=0.027), being married (OR=1.87; [1.40, 2.50], p<0.001), and greater level of social activities (OR=1.05; [1.00, 1.10], p=0.034) were associated with greater comfort with having one’s location tracked while driving by a primary support person, while living alone was associated with lower comfort (OR=0.54; [0.39, 0.75], p<0.001). Women were significantly less likely than men to report comfort with having their audio recorded (OR=0.56; [0.43, 0.73], p<0.001). As with location tracking, those who were married were more comfortable (OR=1.55; [1.18, 2.04], p=0.001) and those who live alone were less comfortable with audio recording (OR=0.61; [0.45, 0.83], p=0.002). In bivariate analysis, no characteristics differentiated respondents by comfort with video chat on wheels during pandemic times, but during normal circumstances, women (OR=0.72; [0.55, 0.93], p=0.011), people with a college degree (OR=0.70; [0.50, 0.97], p=0.033), and those living alone (OR=0.73; [0.54, 1.00], p=0.049) were significantly less comfortable with video chat on wheels. People reporting high confidence using the computer (OR=1.52; [1.07, 2.14], p=0.019) and those with a parent with a history of dementia (OR=1.33; [1.00, 1.76], p=0.050) reported greater comfort levels.

In adjusted, multivariate analysis, higher age (OR=1.02; [1.01, 1.03], p=0.006) and being married (OR=1.66; [1.01, 2.70], p=0.043) remain significant for comfort with having one’s location tracked while driving by a primary support person. Women remain significantly less likely than men to report comfort with having their audio recorded (OR=0.58; [0.43, 0.78], p<0.001). As with bivariate results, no characteristics differentiated respondents by comfort with video chat on wheels during pandemic times. Lower comfort during normal circumstances remains significant for women (OR=0.72; [0.54, 0.97], p=0.030), and those with greater education. Both people with a college degree (OR=0.65; [0.45, 0.94], p=0.023) and those with a master’s degree or higher (OR=0.62; [0.43, 0.89], p=0.009) reported lower comfort with video chat on wheels during normal times. As with bivariate analysis, people reporting high confidence using the computer (OR=1.60; [1.09, 2.35], p=0.017) and those with a parent with a history of dementia (OR=1.46; [1.08, 1.98], p=0.015) reported greater comfort levels with video chat on wheels in an adjusted model.

### 4.2 The importance of five control options

Each of the five options that enable different aspects of control over how technology is used in one’s care were rated by the vast majority of respondents as very or extremely important (Table 4). For the ability to pause a technology in your home when you want privacy, nearly all (94%) rated that as very or extremely important, with only 2.5% rating it as somewhat important or less. Eighty-eight percent think it is very or extremely important that they be able to control when a video chat on wheels is turned on in their home. Eighty-four percent rated trying out a technology before deciding to keep it as very or extremely important. Seventy-six percent thought it would be very or extremely important to be reminded every now and then about what information a technology collects about them, and 67 percent rated it very or extremely important that their support person check in with them about whether they had changed their mind about using a given technology, with 20 percent rating it somewhat important. A very small minority thought any of these options were very unimportant or not at all important (range of 1.6%−4.8%).

### 4.3 Latent classes for control options

**Figure 1:**
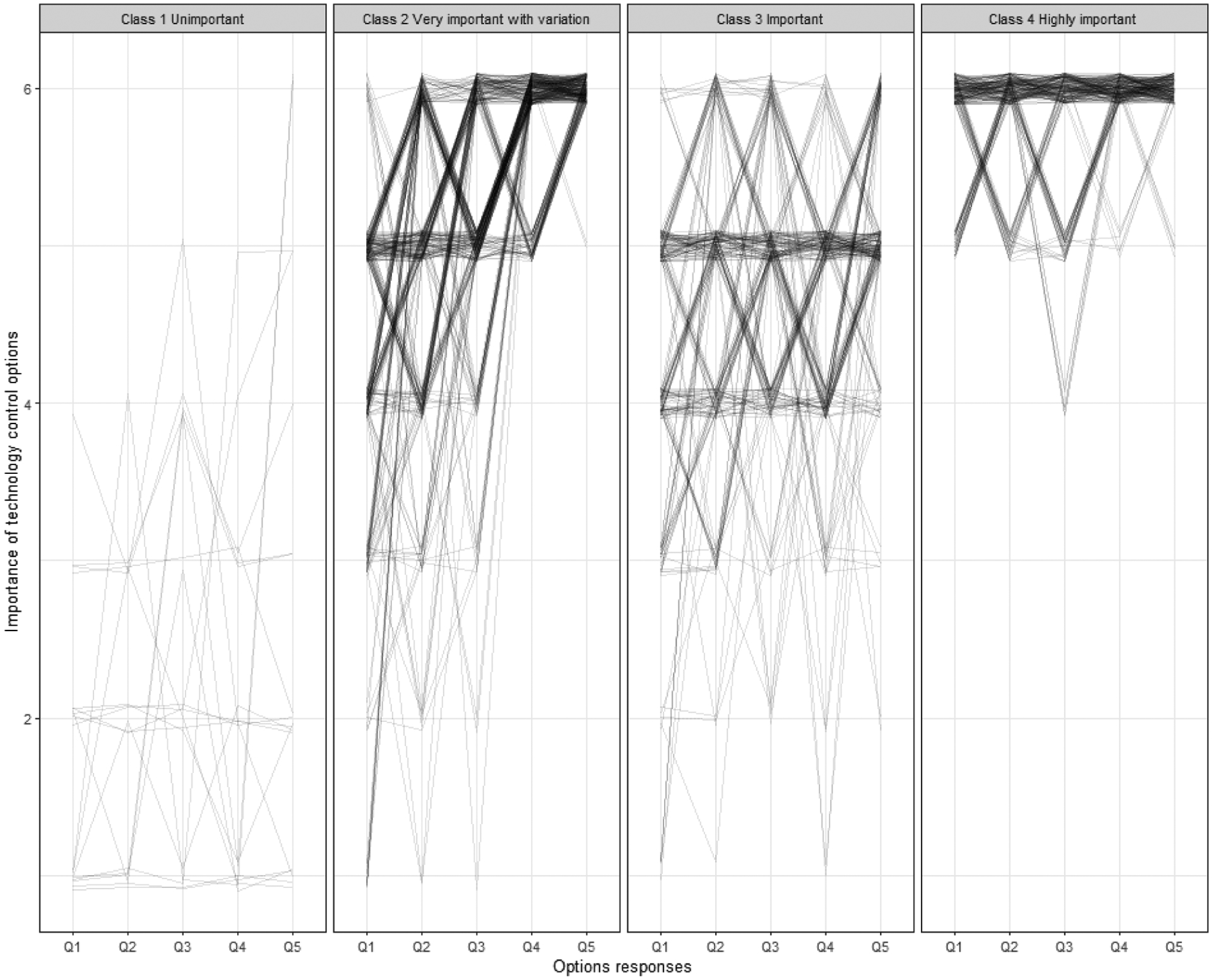
Latent class analysis model: responses stratified by estimated latent class.

Latent class analysis (LCA) was used to understand characteristics predicting how people ranked control on aggregated options. We estimated the number of classes to be four with minimum BIC for LCA. Figure 1 shows the four latent classes relative to the indicator variables describing respondents’ perceived importance of technology control options. Class 1 (n=18, 2.2%) is labeled “Unimportant,” representing a small number of respondents who selected Not at all important to Somewhat unimportant to most questions. Class 2 (n=333, 41.1%) is “Very important with variation.” Respondents reported varied perceived importance, clustering in the very and extremely important range with far lower importance placed on the option of checking in about whether they’ve changed their mind about using the technology. Class 3 (n=232, 28.6%) is labeled “Important” with those who responded to most questions from Somewhat to Very important. Class 4 (n= 227, 28.0%) is labeled “Highly important” with those who selected very or extremely important for most options.

Age, gender, and history of dementia in parents were significantly associated with the four LCA classes (p<0.05) (Table 5). Age tended to be higher in Class 1 of perceiving technology control options to be unimportant compared with the other classes, and Class 4 (Highly important) had lower aged participants compared to Class 3 (Important). Women were overrepresented in Class 4 (Highly important). Participants who had a parent with dementia were less likely to be in class 4 (Highly important) and more likely to be in the small Class 1 (Unimportant). The higher prevalence of memory problem history among respondents in Class 1 compared to all these classes did not reach statistical significance (p=0.120). No significant associations were identified between LCA classes with the other independent variables we examined (marital status, living alone, education, chronic conditions, confidence using computers, and social activity level scores).

## 5 DISCUSSION

Qualitative HCI research has yielded critical insights about barriers to informed use of care technologies, such as lack of feedback and resulting problems of confusion about data flows among older adults. As part of this, researchers have called for the need for privacy options throughout the body of work on remote monitoring technologies for older adults. Robillard has developed stepwise guidelines based on 5 pillars for ethical adoption specifically for dementia care technologies, which begin with participatory design to align needs and outcomes [122]. However, the levers to apply each additional ideal condition for ethical use are often missing. In this context, it is perhaps more critical that design be attentive to user needs.

Our study reveals that nearly three in four survey participants were at least somewhat comfortable using video chat on wheels controlled by a primary support person in their home, and slightly fewer were somewhat comfortable with them tracking their driving location. The least comfort is reported with audio recording to monitor brain health, but still a majority report some comfort with this tracking by a primary support person. A greater majority rated all five control options as very or extremely important, with the ability to pause a device of most importance. An implication of this set of findings on both comfort with monitoring technologies and desire for control options is that technology acceptance studies should assess control (and other) needs in tandem. If we do not understand what needs people expect to be accommodated when they report acceptance, we risk designing based on faulty assumptions and incomplete understanding of their desires and concerns. Below we discuss ways in which design can be responsive to the need for control along with implications for our comparative findings across socio-demographics.

### 5.1 Implications for Design: design’s role in enabling control and privacy options

This study has a number of implications for the design of elder care technologies. It is important to put these in real-world context in which implementation of data intensive technology across care settings can be complicated and vary dramatically depending on specific care dynamics, as well as available resources, whether at the state (i.e., Medicaid) or household level. Policy lags behind use [17, 18, 124] and well-intentioned guidelines to support ethical and more empowering relationships to these technologies lack regulatory power and do not reach into private, familial practices. Codes of conduct and guidelines have been in place for over a decade and yet are still being called for and pointed to as a viable solution to potentially disempowering care practices [6, 118]. Risks are sometimes acknowledged, yet the feedback loop to both design and implementation is not complete [18]. This is likely an effect of consumer focus. That is, products are designed and marketed to caregivers rather to older adults with or on whom they are to be used [145]. As others have pointed out, this is on the one hand unsurprising given the rampant, hidden nature of ageism that manifests in disregard for the need to examine the needs of older adults in technological designs. While the digital divide has been foregrounded in gerontechnology for decades, the concept of “digital ageism” has entered the lexicon, motivated by the discourse on AI harms, particularly regarding race and gender bias [32, 92]. Digital ageism is a result of the youth-centricity of design and technology industries and the often unquestioned stereotypical representation of older adults and old age that permeates many aspects of U.S. cultures [32, 92, 126, 139]. The hidden nature of ageism in the context of limited resources, ethics oversight, and regulation, makes it all the more important for design to foreground issues of power and control.

HCI researchers have investigated issues of power and control of technologies for older adults from the perspectives of different stakeholders. Family members have been found to be willing to support older adults in keeping technology usage secure and private [97]. However, researchers have pointed out that stewardship can sometimes veer into paternalism, where privacy and security decisions are made on behalf of older adults, without their input or even knowledge [107]. Each of the design implications below center the older adult as the primary decision maker and agent in utilizing these options. In doing so, we move the locus of agency towards the older adult, while also recognizing the potential of alternative approaches that harness collective efficacy of communities [76] rather than focusing on individual older adults and designer choices.

#### 5.1.1 To have the ability to pause a technology for privacy.

This research demonstrates strongly that control and privacy options in elder care technologies matter to potential users. Over 90 percent of people felt that the option to pause a technology is very or extremely important. Others have written about the need for this kind of option [96, 145] but none have had the large sample to demonstrate such strong evidence of the desire to have the privacy-enhancing option available. This evidence is needed because the pause option is not typically offered to older adults in technologies to support aging in place or care. Here there is a disconnect between needs and desires and products designed.

Fortunately for design, this is a relatively straightforward solution. Frik et al. have recommended using the most private settings in privacy control defaults to make this option user friendly for older adults [51]. But resistance among companies in this space and in gerontechnology generally to enabling pauses has often been expressed as concern that older adults, and people living with dementia especially, could forget to turn a device back on. Simple design solutions such as an automated timer and indication that the device is re-activating could address this concern. It is worth noting that early emergency alert devices, such as those to detect falls, did not have a cancel emergency response option, despite the high false alert rates and older adults’ preferences to have some control over how a fall is responded to [15]. This trend is changing and more flexible pause and use customization options could follow the same successful route. More work is needed to highlight where the disconnect is in devices on the market as well as in policy and implementation.

#### 5.1.2 To be reminded every now and then about what information a technology collects.

Previous work has found that older adults may be more prone than are younger adults to misperceptions and confusion about data flows. Frik et al. explain:

Data flows in emerging technologies are especially opaque for older adults because they may be less familiar with the state-of-the-art sensors and algorithms, or with advances in artificial intelligence, than the younger population [131]. They may base their assumptions about how devices work—and therefore their privacy mitigations—on analogies with more familiar technology [51:^29^].

Given this problem of misperception, the current study’s insight that older adults generally want to be reminded about what data is collected about them makes evident the need to address awareness. To support boundary management, privacy, and autonomy for a general user population, Leong and Selinger suggest that bots push out reminders that they are not real [83]. A similar approach to enable transparency could be used to signal to older adults what a device is tracking and who is receiving that information. To ensure transparent feedback, devices could visually show the person what information the caregiver is receiving, as suggested by Vines et al. [145] who years ago noted the problem of lack of feedback and resulting misperceptions among older adult study participants. This work would require attentive participatory design to make sure the technique used for such communication is appropriately targeted and successful.

#### 5.1.3 To try out a technology that is used before deciding to keep it and to check in in case preferences change about using the technology.

Eighty-four percent rated the option of trying out a technology first as very or extremely important. Sixty-seven percent rated checkins to learn if they had changed their mind about use as very or extremely important. Others have noted from a neuroethics perspective how consenting is often implicit for elder care technology, leading to recommendations for ongoing, dynamic processes for consenting [123]. Guiding ethical practices that are attentive to this need within private and even publicly funded care provision is a challenge. What role could design play to facilitate this? How can designers and HCI effectively communicate and design for trial periods and routine opportunities to adjust device use? Creative solutions are needed, perhaps including options that embed the norm of trial periods, such as designing a suite of titrated levels of monitoring whereby a user selects the desired option upon testing them in the short term. There is great potential here for designers to influence norms of use, testing, and even ongoing adjustment for personalization. This could be a space for important innovation based on the needs of older adults and the evolving needs of those living with dementia that may be adapted for other user groups.

Standard practices might also be adjusted to accommodate these preferences, in light of the fact that it is hard to fully predict how a technology will be experienced in practice [15]. For example, collaborations could be explored with assistive technology programs, such as U.S. programs funded through the Assistive Technology Act that promote technology access for disabled people, for greater outreach to older adults and caregivers to enable low-stakes trial periods. Business models could offer trial periods or rent to own options that spread payment risk out over time for the consumer or third party payer such that financial commitment is graded to give time to try it out. This would differ from current contract practices. Purchasers might require this to avoid overcommitment to one product solution. In the U.S., this is likely to become a bigger issue with new reimbursement options through cost-conscious third party payers like Medicaid and Medicare for remote patient monitoring and other devices [17, 28]. Moreover, feeling locked into a device can impact relations between beneficiaries and their families and care managers for such programs, placing the frontline workers in difficult positions. Whatever model might be used, it should be based on the expectation that we don’t know how people will interact with a given technology. High initial fees lock people in and should be avoided. These are not design recommendations, per se, but as HCI researchers who have illustrated the complexity of dementia care have pointed out, non-technological features like supports and business models, are also key elements to successfully implemented technology products [148].

#### 5.1.4 To be able to control when a “video chat on wheels” is turned on, if you had one in your home.

Care robots have received heightened attention during the pandemic due to amplified problems among older adults of social isolation [45, 53, 67, 135]. Robots used in elder care range dramatically in data use, integration of AI and form [21, 119, 123]. The technology we posed to study participants falls at one end of this spectrum that does not employ AI as a conversational agent, but rather, as telepresence on wheels in which family and friends could enter a home visually, audibly, and remotely maneuver the location of the robot.

Roaming telepresence like the Beam and Giraff offer the potential benefit of easy use for an older adult with memory or mobility limitations because unlike Zoom, FaceTime, Skype, or other applications, they can be remotely steered [123]. A caregiver could help the person square up to the screen or use the roaming capacity to find the person in their home. The idea that someone could enter another person’s home without need for an acceptance is appealing in emergency situations. But as others have noted, opportunity to accept or decline an initiated telepresence session should be normalized in design and practice [18, 123]. Roaming telepresence without the capability of accepting/declining a call has been cited by gerontechnology domain experts as an extreme violation of personal privacy [18]. For years, the dominant assumption has been that “unobtrusive” technologies with little to no required action on the part of the older adult are ideal, but there are two problems with this assumption. One is that research on actual use has shown how older adults choose to participate as active users and disrupt the “passive age scripts” [108] of devices even when a system is designed for passivity (e.g., passive sensing) [15]. The other issue is that unobtrusive can mean that people are uninformed or unaware and thus lack the privacy they believe they have, and as a consequence cannot adjust their behavior accordingly. Unobtrusiveness implies ease and convenience, which full remote control of telepresence targets; however, older adults are likely to experience visual and audio entrance into the home as invasive if it lacks an option to decline it.

This is not dissimilar from the capacity to pause a monitoring technology when one wants privacy. One can imagine all kinds of scenarios in which it is not a good time for the older adult to have an impromptu visual and audio screen visit from a family member who can control its location in the older adult’s home. The design challenge is how to balance potential need for ease of use and benefits of mobile remote presence with the need for the recipient to have control over initiated calls. This includes accommodating physical difficulty squaring up with or accessing the telepresence robot.

### 5.2 Implications for findings of differences across participants

People ages 65 and older represent multiple generations and age cohorts. They are extraordinarily heterogeneous [90], including in preferences regarding technologies [39] and privacy concerns [14, 51, 128]. The current state of knowledge about the ways these factors may impact user preferences and needs for control options over elder care technology practices is very limited. We included multiple socio-demographic variables in our models and found that age, gender, and experience with dementia in parents are significantly associated with the latent classes for our five control options.

#### 5.2.1 Age.

In adjusted models, age was not associated with comfort with video-chat on wheels or audio recording, but greater age was associated with greater comfort with having one’s location tracked while driving by a primary support person. Like younger participants, most participants over age 65 rated the control options as important; however, older participants were more likely than were younger participants to rate the control options as unimportant or just important and less likely to rate them as very important.

This raises the question, do these control options become less important with age or is this a cohort effect? That is, will the future 65-year-olds of 2050 retain their relative views given cohort-distinct exposures to technology (e.g., through employment vs. retirement exposure) and understandings of risk? Or, longitudinally, do all cohorts of people become slightly inured to lower levels of control over technology as they age? It is possible that greater understanding of a given technology and data flows make one more sensitive to control needs. While we found no significant differences with regard to education among latent classes for the ratings of control, both people with a college degree and those with a master’s degree or more reported lower comfort with video chat on wheels during normal times than did those without a college degree. A recent study of algorithmic awareness in Norway similarly found that negative assessment of algorithmic uses was positively correlated with education, which correlates too with higher awareness of algorithms [55]. Parallel findings about education are described in a survey of Korean older adults where higher education was associated with negative attitudes about sharing a range of personal data with entities including family and hospitals [73].

While our sample was large enough that we could control for covariates, where greater understanding comes from greater exposure, such as through higher formal education or specialized knowledge gained in the workplace, these factors are also associated with age. That is, median higher education attainment increases with new generations, as does tech industry employment, and as such, while we do control for education in our models, age may be confounding for the purpose of distinguishing cohort effect from age. As others have noted in [128], this question of age vs. cohort effect is important for the design community to understand as it seeks to anticipate the technological needs and relevant values held by future older adults. But it is a difficult question to answer given how dramatically technology shifts over time. To understand change over time requires longitudinal studies which are unfortunately rare in this space.

A common question of user research in gerontechnology and HCI in general is “But shouldn’t we expect younger older cohorts to be more accepting and unconcerned with privacy?” Perhaps not necessarily. Our finding about age conflicts with a common assumption that future older adults will be less concerned about privacy and potentially less invested in privacy-enabling control options than are today’s cohorts over age 65. Control options, including pausing for privacy were very important to our entire study sample as a whole, but they were assigned greater weight with lower age. Our participants younger than 65 asserted the importance of these five control options even more strongly than did the older participants. What we can glean from this age-comparative study is that a popular assumption may not be true that today’s younger adults will grow into older adults who are inured to privacy concerns and willing to give up control over data about them. Our study indicates that unless their preferences change over time, they may be even more likely to desire control over technologies used in their care. This will be important to explore through further study as it has implications for how designers should orient to the needs of younger older adults. Regardless, the main takeaway is these control options were very or extremely important to most people across age.

#### 5.2.2 Gender.

In contrast to a recent survey on smart homes that did not find an association between preferences for privacy between demographic traits such as gender and age [10], we found that women were significantly less likely than men to report comfort with having their audio recorded and less comfortable with video chat on wheels during normal times in adjusted models. Our finding that women were less likely to be comfortable with two of the technology scenarios than were men is consistent with research on other areas of data capture and privacy concerns and is particularly meaningful to gerontechnology given the feminization of aging. Women are also overrepresented in the “Highly important” class for the control options. These gender differences might reflect greater vulnerability experienced by women when interacting with monitoring and information technology and associated power dynamics and that these concerns may vary with the technology and purpose. Numerous studies of online privacy concerns suggest that women might experience more risks and concerns compared with men [85]. One explanation is differential experiences with and perceptions of sexual data leakage [77], exploitation, and intimate partner and other abuses (i.e., sexual harassment, doxing, stalking) [93, 99, 133]. Objections, including refusals, that may be partially shaped by experiences of gender-as well as sexual orientation which this study did not examine-will be important to take seriously when engaging older adults of diverse genders in technology that collects and shares private data [33, 118]. Further, the number of men to women sharply declines at older ages, leaving far more women in the 85+ age category, suggesting that designers of care technologies would be wise to attend more to their concerns and needs.

#### 5.2.3 Memory problems and history of a parent with dementia.

Compared with those without a parent with a history of dementia, those with this parental history were significantly more likely to report higher comfort levels with video chat on wheels in an adjusted model. They are also overrepresented among the minority who generally rated the control options as “Unimportant” or just “Important” compared with higher levels of importance. We might infer from these findings that their exposure to dementia and likely caregiver experience also exposed them to the realities of limited tools and resources to provide such care. Given the resource-restricted environment of dementia care in the U.S., it is quite likely that these participants have also experienced under-met need for caregiving supports, memory care, or long-term care for a parent living with dementia. It is probable that witnessing and perhaps providing care to someone through a neurodegenerative disease has helped them to appreciate the challenges of caregiving. That is, their experience may allow them to see greater value in certain tools and place less value on control over these tools in older age. This possibility aligns with previous gerontechnology research that has established that perception of usefulness is a key component for acceptance of technology [41, 82, 143].

There were no statistically significant differences detected between those with self-reported memory problems and those without. Similarly, a longitudinal study about home-based monitoring data and privacy concerns that included people with and without mild cognitive impairment (MCI), found no differences at baseline [24]. They also found few differences between the two groups at one-year follow up. While those without cognitive impairment became significantly less comfortable with video monitoring over the one year study period, people living with MCI did not report a decrease in willingness to be videotaped over time. At one year, participants with MCI were also significantly less likely to report concerns that information could be used to harm them. The authors posit that people with MCI may be more accepting of monitoring due to awareness of threats to their ability to live independently or that they may be less attentive to media content on data and internet risks [24]. Our findings of no difference between people with and without self-reported memory problems suggest that increased perception of health and safety vulnerability that comes with dementia or dementia risk may be counterbalanced by other salient vulnerabilities such as to privacy and autonomy loss, as well as desire not to become a “burden.” New data collection and monitoring may be perceived by different people as alleviating care worry or burden or as intensifying it. One might expect that technologies designed to support people at risk for or living with dementia would be more appealing to people at risk for dementia than those without memory problems. Yet our finding and that of Boise et al. of no greater initial comfort among those experiencing memory problems indicates that other considerations are at play [24].

We found that control over technologies used in care may be no less important to people experiencing memory problems [24]. This aligns with a qualitative study of online safety surveillance for couples with memory concerns in which participants thought that control over settings that would enable personalization would be important [95]. It suggests that an important DIS and HCI path will be to acknowledge and attend to the control needs of people living with dementia over technologies used in their care and to learn how to adjust options that enable control to align with changing capacities. This is a potentially exciting challenge for design that has implications for enabling optimal control for other users of monitoring technologies who have degenerative conditions. Enabling degrees of control with dementia is an important area for further inquiry and more longitudinal research is needed to understand how needs may change over time and with experience gained through actual use.

### 5.3 Limitations and future work

A critical line of inquiry that has to our knowledge not been pursued is to compare the needs of older adults across race and ethnicity, which we were unable to do due the racial homogeneity of our sample. The vast majority of our respondents to this survey were white (95%) and have more formal education and technological experience than the general population and are thus not representative of it. It is possible that the concerns and considerations driving their preferences differ from those of other populations. We cannot speculate at this point given the dearth of research on the potential relationship between race and control needs in care technologies among older adults. There are a couple ways to think about how education level and the high technology access of this online cohort may impact their responses. On the one hand, our study participants’ relative greater access to and comfort with digital technologies likely skewed their responses toward greater comfort and acceptance of the technologies of interest. Because of this, they may represent views closer to the perspective of early adopters. On the other hand, their relative comfort and real-world experience with technology may also give them a stronger understanding of the pitfalls of the technology use scenarios they were presented with (i.e., security issues, privacy threats). Formal education has been associated in other studies and in ours with lower comfort with some forms of data collection, so our sample that has more formal education than the general population may be reporting less comfort and greater control needs than would a representative U.S. sample. It is also important to note that stated opinions about technologies that have not been used do not necessarily predict use, acceptance, or comfort.

Additional factors may have affected the results. Survey respondents were not administered cognitive tests or subjected to physical examinations so that the self-report for memory problems may not accurately reflect actual cognitive performance. The gender variable recorded as part of the initial intake for the online cohort was a limited binary response option of male and female with a third write-in option. For this analysis, we coded binary transgender individuals with their reported gender (those who wrote in trans female (n=1) were coded as women and we coded as men those who wrote in trans male (n=3). Included in the count of missing gender variables are the six additional people who wrote in various responses that broadly fall under the umbrella of gender diverse and questioning because they were excluded from analysis. The exclusion of these six participants is a limitation of this study. Future surveys should provide a broader range of response options for gender and use sampling methods to recruit adequate numbers of participants who identify as non-binary to enable inclusion for multivariate analysis.

Finally, as Nissenbaum has pointed out with regard to reducing privacy protections to control, it is important to note that control is not the end game in ethical, power-aware implementation of technologies that collect, transmit, and analyze new data about older adults [11, 109]. It is possible to achieve informed consent and to enable myriad control options but to do so in a way that results in individual or collective harms [137], such as where choices to use a given technology are still constrained by resource restriction that create unviable or undesirable alternatives to use. That being said, control is important to many older adults and should be extended to contribute to more ethical and equitable practices.

## 6 CONCLUSION

This study builds on previous HCI work by providing direct feedback from potential users on options that could mitigate the primary risks monitoring technologies pose to older adults and care relationships, including those resulting from uninformed use. We presented findings from a survey of 825 people about three technology uses predicted by domain experts to soon be prevalent in dementia home care, followed by five control options. This is the first survey to assess potential users’ interest in such a range of specific options that are within the realm that design can enable. We found that participants report relatively high comfort with sharing data with their primary support person, but that this data collection and sharing is contingent on having control options enabled. All five control options were very or extremely important to most participants, including a full 94 percent for the ability to pause a device. We found no significant difference in adjusted models between those with and without self-reported memory problems for comfort with the technologies or desire for the control options. The control options that matter to the vast majority are not standard options in the design of many products for elder care. We discuss various implications for design to respond to this strong demonstration of user need and argue that design has a significant role to play in enabling more empowering elder care practices. It is important that this role be played given the current limitations to implementing and reinforcing ethics and other guidelines for integrating technologies into elder care practices in ways that protect against risks.

## Supplementary Material

Appendices

## Figures and Tables

**Table 1:** Description of the sample according to all independent variables (n=825)

Category	Subcategories	Mean, SD/Frequencies	Percentage
Age (n=825)	Range: 25–88	Mean=63.93	
		SD=13.17	
Gender (n=825)	Female	534	64.7%
	Male	291	35.3%
Marital status (n=820)	Married/living as if married	577	70.4%
	Not married	243	29.6%
Living status (n=824)	Living alone	162	19.7%
	Living with others	662	80.3%
Education (n=825)	No college degree	202	24.5%
	College degree	276	33.5%
	Master degree and above	347	42.1%
Memory problem history (n=825)	Memory problem reported	201	24.4%
	No memory problem reported	624	75.6%
Number of chronic conditions (n=790)	3+	540	68.4%
	0–2	250	31.6%
Confidence using computer (n=792)	Highly confident	668	84.3%
	Low-moderately confident	124	15.7 %
History of dementia in parents (n=750)	History of dementia in either of parents	226	30.1%
	No history of dementia in either of parents	524	69.9%
Social activity level score (n=800)	Range: 0–17 (out of 20)	Mean:8.47	
		SD=2.82	

**Table 2:** Comfort with four technology scenarios

Survey Items	Response Frequencies			
	Very Uncomfortable n (%)	Somewhat Uncomfortable n (%)	Somewhat Comfortable n (%)	Very Comfortable n (%)
Your primary support person is concerned about your well-being. They want to track where you are when you are driving to make sure you are safe. How comfortable are you with this? (n=789^a^)	91 (11.5%)	137 (17.4%)	234 (29.7%)	327 (41.4%)
New technology that tracks speech changes over time could help people learn about changes to their brain health early. This would allow a person to get help from a medical provider if they have early signs of dementia or memory loss. Your primary support person wants to record audio in your home to learn if and when you might be experiencing changes in your brain health. How comfortable are you with this? (n=825)	154 (18.7%)	193 (23.4%)	245 (29.7%)	233 (28.2%)
Some forms of technology allow a loved one to be a remote presence through video chat (such as FaceTime or Zoom). Unlike those options on your phone or computer, robotic devices are able to be driven remotely in your home. Video chat or “check-in on wheels” can take place anywhere in your home.				
Please think about unusual times when someone cannot come to your home such as during the coronavirus pandemic. In these times, how comfortable would you be with this video chat or “check-in on wheels” driven by your primary support person in your home? (n=825)	88 (10.7%)	132 (16.0%)	293 (35.5%)	312 (37.8%)
Now please imagine that we are again living under normal circumstances so that you are able to spend time in person with other people. In normal times, how comfortable would you be with this video chat or “check-in on wheels” driven by your primary support person in your home? (n=825)	149 (18.1%)	164 (19.9%)	276 (33.5%)	236 (28.6%)

a Missing observations for this question=36 (Not applicable because participants selected that they do not drive)

**Table 3:** Statistically significant variables for bivariate and multivariate ordinal logistic regression

Predictors	Location tracking for driving	Audio recording for brain health	Check-in on wheels during pandemic	Check-in on wheels during normal times
Predictors based on bivariate ordinal logistic regression	Age: 1.01 (1.00–1.02)*	Female: 0.56 (0.43–0.73)***		Female: 0.72 (0.55–0.93)*
	Married/living as if married: 1.87 (1.40–2.50)***	Married/living as if married: 1.55 (1.18–2.04)**		College degree: ^a^ 0.70 (0.50–0.97)*
	Living alone: 0.54 (0.39–0.75)***	Living alone: 0.61 (0.45–0.83)**		Living alone: 0.73 (0.54–1.00)*
	Social activity: 1.05 (1.00–1.10)*			High confidence using computer:^b^ 1.52 (1.07–2.14)*
				History of dementia in parents: 1.33 (1.00–1.76)*
Predictors based on multivariate ordinal logistic regression	Age: 1.02 (1.01–1.03)**	Female: 0.58 (0.43–0.78)***		Female: 0.72 (0.54–0.97)*
	Married/living as if married: 1.66 (1.01–2.70)*			College degree: 0.65 (0.45–0.94)*
				Master degree or above: 0.62 (0.43–0.89)**
				High confidence using computer: 1.60 (1.09–2.35)*
				History of dementia in parents: 1.46 (1.08–1.98)*

a Reference group: No college degree

b Reference group: Low-moderate confidence of using computers

* p<0.05;

** p<0.01;

*** p<0.001

**Table 4:** Frequencies for options responses

	Not at all Important	Very Unimportant	Somewhat Unimportant	Somewhat Important	Very Important	Extremely Important
Q1: To have your primary support person check in with you now and then about whether you’ve changed your mind about using the technology	22 (2.7%)	17 (2.1%)	63 (7.8%)	164 (20.2%)	332 (41.0%)	212 (26.2%)
Q2: To be reminded every now and then about what information a technology collects about you	9 (1.1%)	15 (1.9%)	39 (4.8%)	131 (16.2%)	287 (35.4%)	329 (40.6%)
Q3: To try out a technology that is used in your care before deciding to keep it	6 (0.7%)	12 (1.5%)	17 (2.1%)	95 (11.7%)	326 (40.2%)	354 (43.7%)
Q4: To be able to control when a “video chat on wheels” is turned on, if you had one in your home	9 (1.1%)	7 (0.9%)	15 (1.9%)	67 (8.3%)	207 (25.6%)	505 (62.3%)
Q5: To have the ability to pause a technology in your home when you want privacy	5 (0.6%)	8 (1.0%)	7 (0.9%)	28 (3.5%)	163 (20.1%)	599 (74.0%)

**Table 5:** LCA and independent variables

	Class 1 Unimportant(n=18)	Class 2 - Varied(n=333)	Class 3 - Important(n=232)	Class 4 - Very important(n=227)	P-value
Age	69.1 (11.7)	62.5 (13.1)	66.1 (12.3)	63.3 (13.8)	0.003
Female	66.7%	64.6%	57.3%	72.7%	0.007
Married/living as if married	66.7%	71.5%	70.7%	66.1%	0.503
Living alone	16.7%	18.9%	17.7%	23.8%	0.376
College degree	27.8%	33.6%	33.2%	34.4%	0.514
Master’s degree	38.9%	44.4%	42.7%	37.0%	-
Memory problem history	38.9%	25.2%	19.4%	26.0%	0.120
3+ chronic conditions	66.7%	64.9%	68.1%	63.9%	0.802
High confidence of using computer	61.1%	83.5%	80.6%	79.7%	0.1681
History of dementia in parents	38.9%	25.2%	26.0%	19.4%	0.022
Social activity level score	7.7(2.1)	8.4 (2.9)	8.4 (2.8)	8.7 (2.8)	0.276

## CCS CONCEPTS

• :; • **Human-centered computing** → Human computer interaction (HCI); Empirical studies in HCI; • **Social and professional topics** → User characteristics; Age; Seniors; User characteristics; People with disabilities;

## Footnotes

1 As shown in Appendix A.1, the survey introduction defines primary support person as follows: Your “primary support person” is someone who would be most likely to step in if you needed care or help. We know you may not have a primary support person now but please think about it in terms of your family member or friend who would care for and look out for you.

2 A description of the original RITE Online Cohort, launched in 2015, can be found in Kabacińska et al., 2020.
