# Supplementary material for: Control Matters in Elder Care Technology: Evidence and Direction for Designing It In
Source: DIS (Des Interact Syst Conf). Author manuscript; Available in PMC 2022 Aug 11. (PMC9367632; doi:10.1145/3532106.3533471)
Supplement: Appendices [file NIHMS1827071-supplement-Appendices.docx]

A  APPENDICES

A.1 Survey: RITE: Technology for In-Home Care

Intro: These questions ask you about some new technologies used in home care. The questionnaire should take no more than 10 minutes of your time. We are interested in the opinions of people of all ages, regardless of your experience or lack of experience with care. Some of the questions will ask you to think of your primary support person. Your “primary support person” is someone who would be most likely to step in if you needed care or help. We know you may not have a primary support person now but please think about it in terms of your family member or friend who would care for and look out for you.

q1 Your primary support person is concerned about your well-being. They want to track where you are when you are driving to make sure you are safe.

How comfortable are you with this?

- Very Uncomfortable (1)
- Somewhat Uncomfortable (2)
- Somewhat Comfortable (3)
- Very Comfortable (4)
- Not applicable because I don’t drive (0)

q2 New technology that tracks speech changes over time could help people learn about changes to their brain health early. This would allow a person to get help from a medical provider if they have early signs of dementia or memory loss. Your primary support person wants to record audio in your home to learn if and when you might be experiencing changes in your brain health.
How comfortable are you with this?

- Very Uncomfortable (1)
- Somewhat Uncomfortable (2)
- Somewhat Comfortable (3)
- Very Comfortable (4)

q3 Some forms of technology allow a loved one to be a remote presence through video chat (such as FaceTime or Zoom). Unlike those options on your phone or computer, robotic devices are able to be driven remotely in your home. Video chat or "check-in on wheels" can take place anywhere in your home. Examples of these devices are shown below.

Images retrieved from https://robots.nu/en/robot/giraff-telepresence-robot and <https://suitabletech.com/>

q3_a Please think about unusual times when someone cannot come to your home such as during the coronavirus pandemic. In these times, how comfortable would you be with this video chat or "check-in on wheels" driven by your primary support person in your home?

- Very Uncomfortable (1)
- Somewhat Uncomfortable (2)
- Somewhat Comfortable (3)
- Very Comfortable (4)

q3_b Now please imagine that we are again living under normal circumstances so that you are able to spend time in person with other people.
In normal times, how comfortable would you be with this video chat or "check-in on wheels" driven by your primary support person in your home?

- Very Uncomfortable (1)
- Somewhat Uncomfortable (2)
- Somewhat Comfortable (3)
- Very Comfortable (4)

Q8 Please think about technologies in general that allow a primary support person to monitor a care recipient remotely, like those you just answered questions about. How important would each of the following options be to you:

|  | Not at all Important | Very Unimportant | Somewhat Unimportant | Somewhat Important | Important | Extremely Important |
| --- | --- | --- | --- | --- | --- | --- |
| To try out a technology that is used in your care before deciding to keep it   (1) | O | O | O | O | O | O |
| To be reminded every now and then about what information a technology collects about you   (2) | O | O | O | O | O | O |
| To have your primary support person check in with you now and then about whether you’ve changed your mind about using the technology   (3) | O | O | O | O | O | O |
| To be able to control when a “video chat on wheels” is turned on, if you had one in your home   (4) | O | O | O | O | O | O |
| To have the ability to pause a technology in your home when you want privacy   (5) | O | O | O | O | O | O |

A.2  Latent class analysis for ordinal responses statistic report

Since the interest lies in learning underlying latent classes of participants, we conducted a latent class analysis model (LCA). The basic idea of the latent class model is that the variability in the responses can be explained by a few underlying latent classes, which are unknown but can be estimated from the data. Let us assume there are $R$ latent classes. These sub-groups or *classes* often involve participants that respond to questions in a similar pattern.

We used a latent class analysis model to capture underlying patterns in observed data. We evaluated if there were certain demographic variables that were associated with estimated latent class using statistical tests of association.

We started by describing the statistical model. Let us denote that $N=810$ participants respond to $J=5$ questions each with $K_{j}=K=6$responses. Let $Y_{ijk}=1$ if $i$th participant responds to $j$th question with response $k$ and $Y_{ijk}=0$, otherwise. For example, if a participant $5$ responds $(3)$ somewhat unimportant to Q2, then, $Y_{i=5,j=2,k=3}=1$ and $Y_{i=5,j=2,k=k^{'}}=0$ for $k^{'}\neq3$.

Let us assume that there are $R$ latent classes. Let $Z_{i}$ be a latent variable which takes values in $\{1,\ldots,R\}$ and indicate the latent class membership of participant $i$. Let $\pi_{jrk}=Pr\left( Y_{jk}=1 | Z_{i}=r \right)$ denote the latent class conditioned probability. It is the probability that a participant responds $k$ to the $j$th question given that the participant belongs to $r$th cluster. Note that this class conditioned probability sums to 1 over all possible responses, i.e. $\sum_{k=1}^{K} \pi_{jrk}=1$. Let $p_{r}$ denote probability that a participant belongs to cluster $r$.

Latent class models typically assume that question responses are independent given the underlying latent class. This implies that, given the latent class for a participant $i$, we can write the conditional likelihood as follows:

$$f\left( Y_{i} | Z_{i}=r,\pi\right)=\prod_{j=1}^{J} \prod_{k=1}^{K} \pi_{jrk}^{Y_{ijk}}.$$

And hence the marginal likelihood of the responses for a participant $i$ is given by

$$f\left( Y_{i} | \pi,p \right) = \sum_{r=1}^{R} P\left( Z_{i}=r \right)f\left( Y_{i} | Z_{i}=r,\pi\right)$$

$$=\sum_{r=1}^{R} p_{r}f\left( Y_{i} | Z_{i}=r,\pi\right)$$

$$=\sum_{r=1}^{R} p_{r}\prod_{j=1}^{J} \prod_{k=1}^{K} \pi_{jrk}^{Y_{ijk}}.$$

Hence, the log-likelihood of responses by $N$ participants is given by

(1)

$$\log L=\log f\left( Y_{1},Y_{2},\ldots,Y_{N} | \pi,p \right)=\sum_{i=1}^{N} \log\sum_{r=1}^{R} p_{r}\prod_{j=1}^{J} \prod_{k=1}^{K} \pi_{jrk}^{Y_{ijk}}.$$

Note that some existing packages including poLCA [1] performs LCA for polytomous nominal categorical variables. poLCA treats ordinal categorical variables as nominal categorical variables and claims that there is no practical difference treating ordinal variables as nominal. However, treating ordinal variables as nominal excludes potentially useful information contained those variables [2, pp. 2, Section 1.2, 3].

We modeled the ordinal nature of responses using an adjacent category logit model [3] [2, pp. 90, Section 4.1, Eq. 4.4]. In particular, we used the row effects model, first proposed by [4] to model the local log-odds of a response $k$ for question $j$ as:

$$\log\left( \frac{\pi_{jr,k}}{\pi_{jr,k+1}} \right)=\alpha_{jk}+\tau_{jr}\quad k=1,\ldots,K-1;r=1,\ldots,R;j=1,\ldots J.$$

This model specifies a common effect $\alpha_{jk}$ across latent classes for each response level and question. Additionally, it specifies a latent class effect $\tau_{jr}$ which can be thought of as a class specific random effect that modifies the local odds of response level $k$ vs. $k+1$ by $\tau_{jr}$. Note that in poLCA [1], each $\pi_{jrk}$ is modelled separately. Modelling the ordinal structure as above allows to produce a more parsimonious model for class-conditional probabilities. Note that $\tau_{jR}=0$ for all $j$ for identifiability reasons and we still have the constraint that $\sum_{k=1}^{K} \pi_{jrk}=1$.

Using the above definition of local log odds, it can be shown that the $\pi_{jrk}$ are:

$$\pi_{jrk}=\frac{\exp\left( \sum_{k^{'}=k}^{K-1} \alpha_{jk^{'}}+\left( K-k \right)\tau_{jr} \right)}{1+\sum_{l=1}^{K-1} \exp\left( \sum_{k^{'}=l}^{K-1} \alpha_{jk^{'}}+\left( K-l \right)\tau_{jr} \right)};$$

$$\pi_{jrK}=\frac{1}{1+\sum_{l=1}^{K-1} \exp\left( \sum_{k^{'}=l}^{K-1} \alpha_{jk^{'}}+\left( K-l \right)\tau_{jr} \right)}.$$

Plugging the above class conditional probabilities in (1) gives us the joint likelihood of ordinal responses as a function $\alpha,\tau,p$ :

$$\log L=\log f\left( Y_{1},Y_{2},\ldots,Y_{N} | \alpha,\tau,p \right)=\sum_{i=1}^{N} \log\sum_{r=1}^{R} p_{r}\prod_{j=1}^{J} \prod_{k=1}^{K} \pi_{jrk}^{Y_{ijk}}$$

where $\pi_{jrk}$ are function of $\alpha,\tau$ as shown above.

Note that this model accounts for the ordinal nature of response variables while keeping the latent class structure intact. Additionally, this model is more parsimonious than LCA model in poLCA since the number of parameters is $\sum_{j} \left( K-1 \right)+\left( J+1 \right)\left( R-1 \right)$ as opposed to poLCA package where the number of parameters is $R\sum_{j} \left( K-1 \right)+\left( R-1 \right)$.

A.2.1 Parameter estimation

We estimated parameters $p_{r},\alpha_{jk},\tau_{jr}$ using an Expectation Maximization (EM) approach [5]. In it, we estimated the parameters iteratively, alternately using an expectation step to update latent class membership probability $p_{r}$ and a maximization step to update $\alpha,\tau$.

Given estimates $\hat{\alpha},\hat{\tau}$ and hence $\hat{\pi},$we updated the probability of each participant belonging to latent class $r$ using Bayes theorem as follows:

$$\hat{P}\left( Z_{i}=r | Y_{i} \right)=\frac{\hat{P}\left( Y_{i} | Z_{i}=r,\hat{\pi} \right)\hat{P}\left( Z_{i}=r \right)}{P\left( Y_{i} | \hat{\pi},\hat{p} \right)}=\frac{\hat{p_{r}}f\left( Y_{i} | Z_{i}=r,\hat{\pi} \right)}{\sum_{r=1}^{R} \hat{p_{r}}f\left( Y_{i} | Z_{i}=r,\hat{\pi} \right)}$$

Using these probabilities, in the maximization step, we updated $\hat{p_{r}}$ as

$$\hat{p_{r}^{new}} = \frac{1}{N} \sum_{i=1}^{N} \hat{P}\left( Z_{i}=r | Y_{i} \right) \quad i=1,\ldots,N.$$

The maximization step also involved updating $\alpha,\tau$ using a one step gradient descent algorithm with a learning parameter $\eta$ ([link](https://bit.ly/3x7Iuhq)). We used gradient descent because values of $\alpha,\tau$ that maximize $\log L$ are not available in closed form. Gradient descent is a numerical optimisation algorithm used to find the local maximum by making small steps (controlled by $\eta$) in the direction opposite to the gradient. The update equation for $\alpha,\tau$ is given by:

$$\left( \begin{matrix} \hat{\boldsymbol{\alpha}^{new}} \\ \hat{\boldsymbol{\tau}^{new}} \end{matrix} \right)=\left( \begin{matrix} \hat{\boldsymbol{\alpha}^{old}} \\ \hat{\boldsymbol{\tau}^{old}} \end{matrix} \right)-\eta{\nabla Log L|}_{\left( \alpha^{old},\tau^{old} \right)}$$

where

$$\frac{\partial\log L}{\partial\alpha_{jm}}=\sum_{r=1}^{R} \sum_{i=1}^{N} \sum_{l=1}^{m} \hat{p_{ir}}\left( Y_{ijl}-\pi_{jrl} \right)$$

$$\frac{\partial\log L}{\partial\tau_{jq}}=\sum_{i=1}^{N} \hat{p_{ir}}\left[ \sum_{k=1}^{K} \left( K-k \right)\left( Y_{ijk}-\pi_{jqk} \right) \right]$$

for $m=1,\ldots,K-1, q=1,\ldots,R-1, j=1,\ldots, J$. We used $\eta= 0.001$ as the fixed learning rate in our analysis. Hence, to summarize, our estimation procedure worked as follows:

- (Set t=0) Obtain initial estimates for $p,\alpha,\tau$. We initialize $p_{r}$ uniformly, i,e, $p_{r}=1/R$ and we initialize $\alpha$'s and $\tau$'s to be drawn from normal(0,2) and normal(0,1) respectively. $\alpha$'s and $\tau$'s can be real numbers and hence the normal initialisation and since class proportions $p_{r}$ are not known a priori, we initialise them uniformly.
- Expectation step: Calculate the latent class membership posterior probabilities at time $t+1$, $\hat{P^{\left( t+1 \right)}}\left( Z_{i}=r | Y_{i} \right)$ using values of $\hat{p_{r}^{\left( t \right)}}$ and $\hat{\pi^{\left( t \right)}}$ calculated using $\hat{\alpha^{\left( t \right)}},\hat{\tau^{\left( t \right)}}$ .
- Maximization step: Calculate

$$\left( \begin{matrix} \hat{\boldsymbol{\alpha}^{\left( t+1 \right)}} \\ \hat{\boldsymbol{\tau}^{\left( t+1 \right)}} \end{matrix} \right)=\left( \begin{matrix} \hat{\boldsymbol{\alpha}^{\left( t \right)}} \\ \hat{\boldsymbol{\tau}^{\left( t \right)}} \end{matrix} \right)-\eta{\nabla Log L|}_{\left( \backslash bold\alpha^{\left( t \right)},\tau^{\left( t \right)} \right)}$$

- Iterate Expectation and Maximization step until convergence

Since EM algorithms could often get stuck in local maxima, we ran the above algorithm multiple times with different starting values and assessed the impact of these starting values on the results.

A.2.2 Results

We implemented the EM algorithm procedure above with 20 random starting values running each for 1000 iterations or until convergence was met. We used BIC to identify the number of latent classes. Let $\log L^{*}$ represents the maximum log likelihood and $\Phi$ denote the total number of parameters, then

$$BIC=-2\log L^{*}+\Phi\log N$$

where in our modelling $\Phi=\sum_{j} \left( K-1 \right)+\left( J+1 \right)\left( R-1 \right)$. We additionally calculated AIC and Pearson's $\chi^{2}$ statistic likelihood ratio chi-square $(G^{2})$ similarly to poLCA package. We selected the number of classes based on which value led to minimum value of BIC. Since we used marginal likelihood of the data, inappropriateness of BIC is not encountered here. BIC was preferred over AIC since it penalized larger models more aggressively and promotes parsimonious models. We estimated the number of classes to be four with minimum BIC for LCA to be 8336.11 (Figure 1).


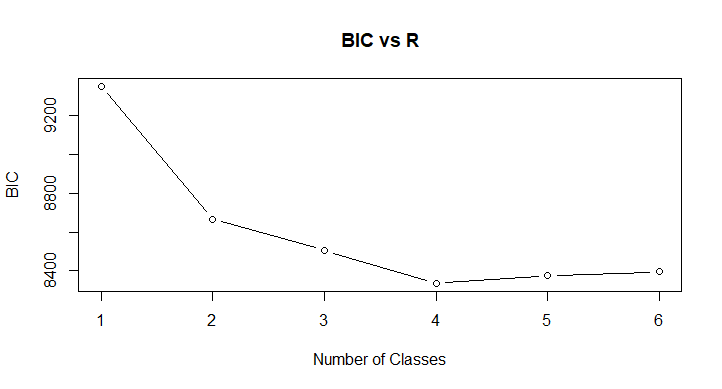


Figure 1: BIC plotted as a function of latent class R

We calculated the posterior latent class membership probabilities, $\hat{P}\left( Z_{i}=r | Y_{i} \right)$, for each subject $i$ and allot person $i$to cluster $r$ with the highest latent class membership probability. We presented here a jittered spaghetti plot of participants stratified by their estimated latent class.


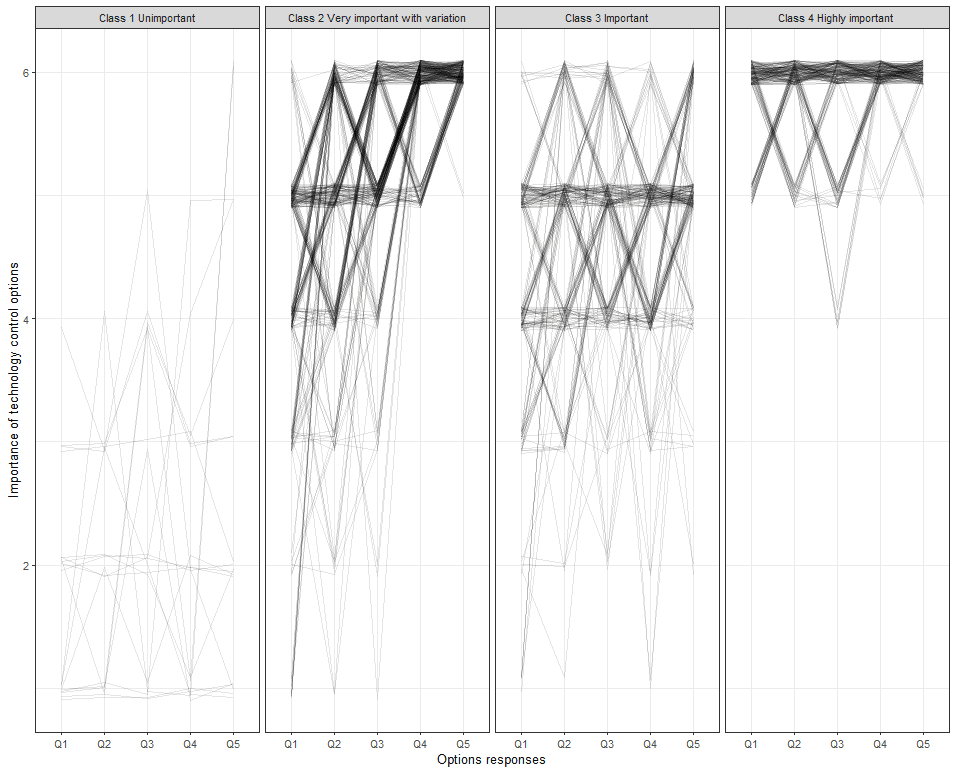


Figure 2: Jittered spaghetti plots of responses stratified by estimated latent class; Each lines represents responses of one participants; Responses are jittered for visibility; Intensity of lines denotes the frequency of that combination of response for five questions

REFERENCES

| 1. [1] Drew A. Linzer and Jeffrey B. Lewis. 2011. poLCA: An R package for polytomous variable latent class analysis. Journal of statistical software 42: 1–29. |
| --- |
| 1. [2] Alan Agresti. 2010. Analysis of Ordinal Categorical Data. John Wiley & Sons. |
| 1. [3] Alan Agresti and Joseph B. Lang. 1993. Quasi-Symmetric Latent Class Models, with Application to Rater Agreement. Biometrics 49, 1: 131–139. <https://doi.org/10.2307/2532608> |
| 1. [4] Gary Simon. 1974. Alternative Analyses for the Singly-Ordered Contingency Table. Journal of the American Statistical Association 69, 348: 971–976. <https://doi.org/10.1080/01621459.1974.10480239> |
| 1. [5] A. P. Dempster, N. M. Laird, and D. B. Rubin. 1977. Maximum Likelihood from Incomplete Data Via the EM Algorithm. Journal of the Royal Statistical Society: Series B (Methodological) 39, 1: 1–22. <https://doi.org/10.1111/j.2517-6161.1977.tb01600.x> |
